# Complete Genome Sequence of Alcaligenes faecalis Phage Piluca

**DOI:** 10.1128/mra.00124-22

**Published:** 2022-03-15

**Authors:** Joshua Wan, Zihao Yu, James Clark, Tram Le, Ryland Young, Mei Liu

**Affiliations:** a Department of Biochemistry and Biophysics, Texas A&M University, College Station, Texas, USA; b Center for Phage Technology, Texas A&M University, College Station, Texas, USA; Queens College CUNY

## Abstract

Alcaligenes faecalis is an opportunistic pathogen exhibiting drug resistance. Here, the 35,451-bp genome of *A. faecalis* phage Piluca is described. Piluca is not closely related to any isolated phages in the NCBI database. Piluca possesses genes encoding CI-like and Cro-like repressors and a tyrosine integrase, suggesting its temperate lifestyle.

## ANNOUNCEMENT

Alcaligenes faecalis is a Gram-negative bacterium commonly found in watery environments and causes opportunistic infections in humans that are difficult to treat because of increased resistance to antibiotics (1, 2). Phages are of interest as potential therapeutic agents to control infections associated with this bacterium.

Phage Piluca was isolated in 2019 from a wastewater sample collected in a wastewater treatment plant in Madisonville, TX, using *A. faecalis* ATCC strain 8750 as the isolation host. The bacterial host strain was cultured using tryptic soy broth or agar (Difco) at 37°C with aeration, and the phage was isolated and propagated using the soft agar overlay method (3). Phage DNA was purified from ∼8 mL phage lysate using the Promega Wizard DNA cleanup system as previously described (4). DNA sequencing libraries were prepared as 300-bp inserts using a Swift 2S Turbo kit and sequenced on an Illumina MiSeq instrument with paired-end 150-bp reads using V2 300-cycle chemistry. This produced 203,894 total raw reads, which were then quality controlled using FastQC (www.bioinformatics.babraham.ac.uk/projects/fastqc) and trimmed with FastX-Toolkit v0.11.6 (http://hannonlab.cshl.edu/fastx_toolkit/). The raw reads were assembled into a single contig using SPAdes v3.5.0 (5) with 244-fold coverage, and the contig sequence was completed by PCR amplifying off the contig ends (primers: forward 5′-CGGCAATGGGAATGCATGAG-3′, reverse 5′-TTGGTGAAAGAATGGCCCGT-′3) and Sanger sequencing of the resulting product followed by manual curation. The genome was annotated using the CPT Galaxy-Apollo phage annotation platform (https://cpt.tamu.edu/galaxy-pub) (6–8). Structural annotation was performed with GLIMMER v3 (9) and MetaGeneAnnotator v1.0 (10). tRNAs were detected using ARAGORN v2.36 (11) and tRNAscan-SE v2.0 (12). Gene functions were predicted with InterProScan v5.48 (13), BLAST v2.9.0 (14), TMHMM v2.0 (15), HHPred (16), LipoP v1.0 (17), and SignalP v5.0 (18). BLAST searches were compared to the NCBI nonredundant (nr) and SwissProt databases (19). Genome-wide DNA sequence similarity was calculated with progressive Mauve v2.4 (20). All tools were run at default settings.

Phage Piluca has a 35,451-bp genome with 41% G+C content and 96.1% coding density. Piluca is not closely related enough to any isolated phages in the NCBI nr database to be placed in a phage type group in the *Podoviridae* family, as it is lacking genome-wide nucleotide identity and shares no more than 12 proteins with any other isolated phage (BLASTp E value, <0.001). Piluca is predicted to be a podophage based on its tail proteins that show significant similarity (BLASTp E value, <0.001) to that carried by *Enterobacteria* podophage T7 (GenBank accession number NC_001604) and phage epsilon15 (NC_004775). Piluca possesses genes encoding CI-like and Cro-like transcriptional regulators and a tyrosine integrase, suggesting it possesses a temperate lifestyle. Piluca is related on the nucleotide level to prophage elements identified in multiple Acinetobacter baumannii strains, such as A. baumannii strain VB82 (CP050385, ∼65% overall similarity based on BLASTn). Overall, 23 of the total 49 predicted protein-coding genes could be assigned a putative function. Piluca has genes encoding a class I holin and a signal-arrest-release (SAR) endolysin, but no spanins were identified. No tRNAs were identified in the genome. PhageTerm (21) analysis of the raw sequencing reads indicated that Piluca packages its DNA by a headfull mechanism.

### Data availability.

The Phage Piluca genome sequence was deposited in GenBank under accession number MZ326864. The associated BioProject, SRA, and BioSample accession numbers are PRJNA222858, SRR14095246, and SAMN18509619, respectively.
